# Supplementary figures and images for: COVID-19 Breakthrough Infections in Vaccinated Kidney Transplant Recipients
Source: Vaccines (Basel). 2022 Nov 11;10(11):1911. doi: 10.3390/vaccines10111911 (PMC9696595; doi:10.3390/vaccines10111911)

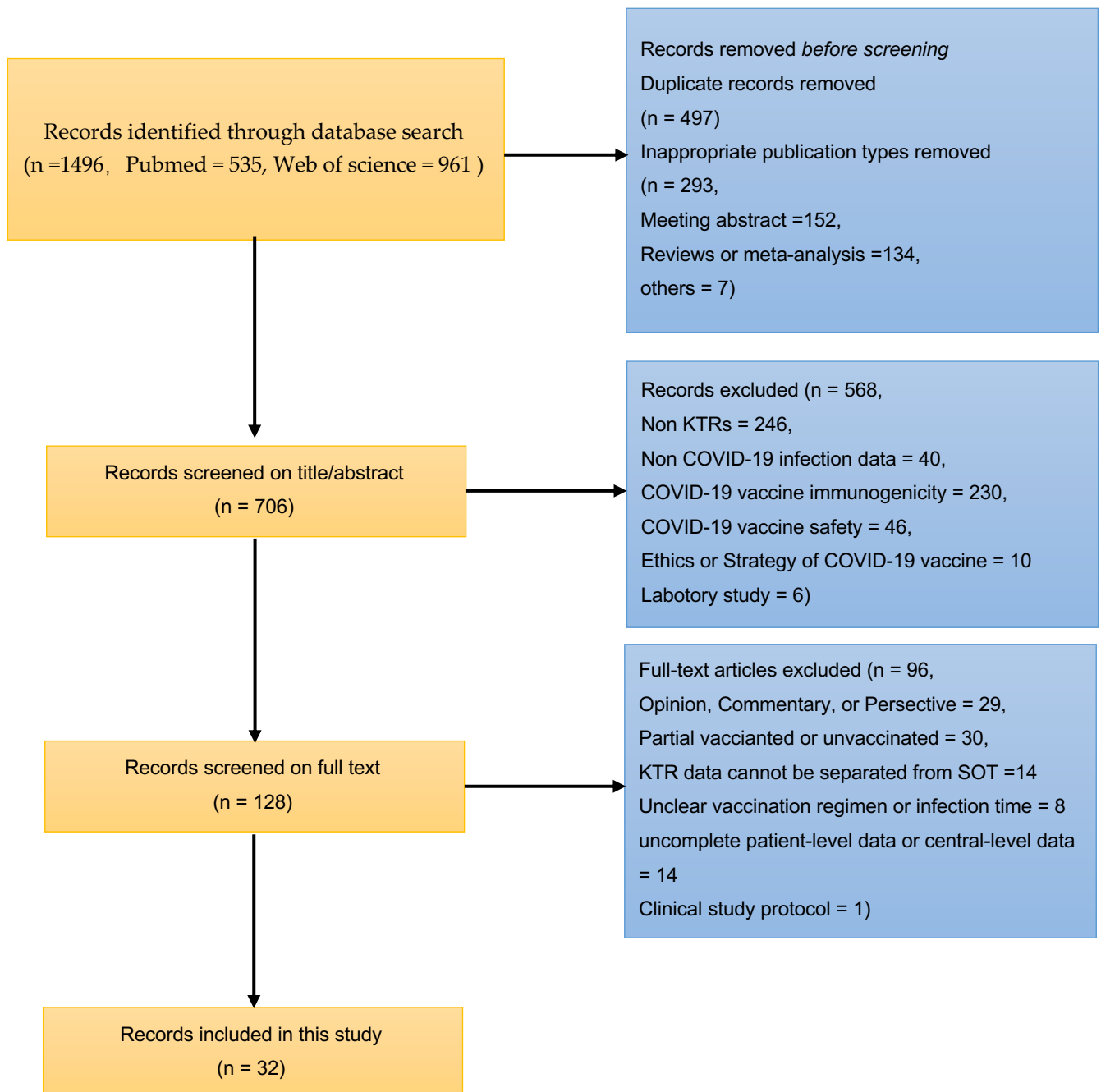

**Fiugure S1.** Flow diagram for study selection.

Supplement: Supplementary file 1 [file vaccines-10-01911-s001.zip › vaccines-1971843-supplementary.pdf]
